# Effects of acute and chronic interval sprint exercise performed on a manually propelled treadmill on upper limb vascular mechanics in healthy young men

**DOI:** 10.14814/phy2.12861

**Published:** 2016-07-12

**Authors:** T. Dylan Olver, Steph M. Reid, Alan R. Smith, Mair Zamir, Peter W. R. Lemon, M. Harold Laughlin, J. Kevin Shoemaker

**Affiliations:** ^1^ Neurovascular Research LaboratorySchool of KinesiologyThe University of Western OntarioLondonOntarioCanada; ^2^ Department of Biomedical SciencesUniversity of Missouri‐ColumbiaColumbiaMissouri; ^3^ Exercise Nutrition LaboratorySchool of KinesiologyThe University of Western OntarioLondonOntarioCanada; ^4^ Departments of Applied Mathematics and of Medical BiophysicsThe University of Western OntarioLondonOntarioCanada

**Keywords:** Arterial stiffness, interval sprint training, vascular mechanics, vascular remodeling

## Abstract

Interval sprint exercise performed on a manually propelled treadmill, where the hands grip the handle bars, engages lower and upper limb skeletal muscle, but little is known regarding the effects of this exercise modality on the upper limb vasculature. We tested the hypotheses that an acute bout of sprint exercise and 6 weeks of training induces brachial artery (BA) and forearm vascular remodeling, favoring a more compliant system. Before and following a single bout of exercise as well as 6 weeks of training three types of vascular properties/methodologies were examined in healthy men: (1) stiffness of the entire upper limb vascular system (pulse wave velocity (PWV); (2) local stiffness of the BA; and (3) properties of the entire forearm vascular bed (determined by a modified lumped parameter Windkessel model). Following sprint exercise, PWV declined (*P* < 0.01), indices of BA stiffness did not change (*P* ≥ 0.10), and forearm vascular bed compliance increased and inertance and viscoelasticity decreased (*P* ≤ 0.03). Following manually propelled treadmill training, PWV remained unchanged (*P* = 0.31), indices of BA stiffness increased (*P* ≤ 0.05) and forearm vascular bed viscoelasticity declined (*P* = 0.02), but resistance, compliance, and inertance remained unchanged (*P* ≥ 0.10) compared with pretraining values. Sprint exercise induced a more compliant forearm vascular bed, without altering indices of BA stiffness. These effects were transient, as following training the forearm vascular bed was not more compliant and indices of BA stiffness increased. On the basis of these data, we conclude that adaptations to acute and chronic sprint exercise on a manually propelled treadmill are not uniform along the arterial tree in upper limb.

## Introduction

Increases in skeletal muscle blood flow at the onset of exercise are coupled to mechanical work (Saltin et al. 1998), occur rapidly (Shoemaker et al. 1997, 1999a,b; VanBeekvelt et al. 2001), and are achieved and maintained by a combination of mechanical and metabolic stimuli (Clifford and Hellsten 2004; Clifford 2007). Chronically repeated bouts of exercise (i.e., exercise training) result in structural vascular (i.e., angiogenesis, remodeling of arteries, and arteriogenesis), and functional (i.e., altered control of vascular resistance) adaptations (Laughlin and Roseguini 2008; Laughlin et al. 2012). During interval sprint exercise (characterized by 4 × 30 sec “all‐out” efforts separated by a ~4 min recovery) participants exert maximal effort, and obtain near maximal VO_2_ (~90%) and heart rate (~90%) values as well as significant deoxygenation in the *vastus lateralis* (Buchheit et al. 2012; Hazell et al. 2014). Indeed, chronic interval sprint training, is a potent stimulus that increases microvascular eNOS content in the *vastus lateralis* (Cocks et al. 2013) and improves conduit artery vascular endothelial function in both the lower and upper limb, indicated by augmented flow‐mediated dilation in the popliteal and brachial arteries (BA) (Rakobowchuk et al. 2008; Harris et al. 2014). Furthermore, bouts of interval sprint exercise and training alter lower limb vascular structure, indicated by an acute reduction in lower limb pulse wave velocity (PWV)(Rakobowchuk et al. 2009) following bouts of exercise and an increase in popliteal distensibility and capillary density in *vastus lateralis* following chronic training (Rakobowchuk et al. 2008; Cocks et al. 2013). Whether acute bouts of interval sprint exercise or exercise training conducted on a manually propelled treadmill or cycle ergometer induce vascular remodeling in the BA and forearm vasculature remains to be elucidated.

In contrast to interval sprint exercise performed on a track or a motorized treadmill, interval sprint exercise performed on a cycle ergometer or a manually propelled treadmill engages the forearm skeletal muscle because the hands grip the handle bars isometrically at intensities up to 100% of maximum voluntary contraction (T.D. Olver, A.R. Smith unpublished observation – pilot testing on 3 subjects in our lab revealed that maximum voluntary hand grip strength was ~9% greater during a bout of sprint exercise versus in a seated position, not performing whole‐body exercise). Importantly, isometric handgrip training alone can improve forearm reactive hyperemia, indicative of microvascular remodeling (Takeshita and Mark 1980), without altering endothelial function (Green et al. 1994). Little is known regarding the impact of acute and chronic interval sprint exercise on BA and forearm vascular remodeling. Furthermore, the impact of interval sprint training conducted on a manually propelled treadmill or cycle ergometer on the properties of conduit versus arteriolar levels of the vascular bed in the upper limb have not been studied. Given that age, hypertension, and smoking status impact the upper limb vasculature along the arterial tree (i.e., at the conduit and arteriolar level) (Bulpitt et al. 1999; van der Heijden‐Spek et al. 2000; Zamir et al. 2009; Nielson et al. 2014), determining if acute or chronic sprint exercise induces vascular remodeling in the upper limb could provide insight into the potential therapeutic efficacy of this type of training. Therefore, the purpose of this study was to determine whether vascular adaptation occurs, and to characterize changes in various segments of the vascular bed in the upper limb in response to an acute bout of sprint exercise and after 6 weeks of training performed on a manually propelled treadmill. Consistent with observations in the conduit arteries and microcirculation of the lower limb/prime movers (Rakobowchuk et al. 2008, 2009; Cocks et al. 2013) this study tested two hypotheses: Hypothesis 1, that a single bout of interval sprint exercise induces vascular mechanical adaptations, favoring a more compliant upper limb vascular system, at the level of the conduit artery and entire forearm vascular bed and Hypothesis 2, that 6 weeks of exercise training conducted on a manually propelled treadmill would also induce vascular mechanical adaptations, favoring a more compliant upper limb vascular system, at the level of the conduit artery and entire forearm vascular bed.

In this study, we employ three methods to study vascular mechanical indices in three vascular segments: (1) the entire upper limb using PWV (the speed that the arterial pulse propagates from the aortic arch to the finger, indicative of stiffness along the upper limb vasculature) (Nichols 2005); (2) the local conduit artery using measures of vascular stiffness (direct measure of diastolic and systolic arterial diameter and pressure at the same site) (O'Rourke et al. 2002); and (3) the entire forearm vascular bed, using a three‐element lumped Windkessel model (mechanical properties of the oscillatory flow and pressure waveform at the point of entry of the vascular bed) (Zamir et al. 2009). Although these measures interact (i.e., PWV is determined, in part, by BA and forearm vascular mechanics); changes can occur independently in each segment as evaluated by the model. Thus, we treat these parameters as independent measures of adaptations of vascular structure/function occurring throughout the entire upper limb, in the conduit artery/BA alone and in the forearm vascular bed (including microvasculature), respectively (Nielson et al. 2014). In this regard, because the vascular properties assessed using each model overlap to some extent, but also operate independently we are able to form inferences on the nature and specific location of exercise‐induced upper limb vascular adaptations. Harris and colleagues (Harris et al. 2014) reported that upper limb PWV does not change following interval sprint training performed on a cycle ergometer. Thus, consistent with observations in conduit arteries and the microcirculation of the lower limb (Rakobowchuk et al. 2008, 2009; Cocks et al. 2013), we believe that chronic structural vascular adaptations to interval sprint training performed on a manually propelled treadmill will be most evident in the BA and the forearm vascular bed versus in a whole upper limb grouped model (i.e., PWV).

## Methods

### Subjects

Sixteen recreationally active (nonsystematically trained and not involved in a regimented exercise training program) male subjects participated in this study. All experimental procedures and potential risks were explained fully to the subjects prior to any testing and subjects provided written, informed consent. Health status was assessed using the Physical Activity Readiness Questionnaire (Thomas et al. 1992). This study was approved by the University of Western Ontario Ethics Committee for Research on Human Subjects.

### Study design

Subjects abstained from alcohol (24 h), caffeine (24 h) and exercise (48 h) prior to data collection. They arrived to the laboratory at least 4 h after their last meal and body composition was measured (air‐displacement plethysmography). Thereafter, the subjects laid supine on a tilt table for 15 min prior to baseline hemodynamic measures. Then, BA and forearm vascular mechanics were examined before (pre‐) and 20 min after one acute bout of sprint exercise (*n* = 8) or after 6 weeks of training (*n* = 8). Subjects were familiarized with all testing and exercise procedures prior to baseline measures. In the sprint exercise protocol, measurements were made immediately before and ~20 min following the acute bout of exercise. In the training protocol, measures were made a day before the first training session and ~48 h following the last training session at the same time of day and under the same conditions in order to avoid confounding effects.

### Interval sprint exercise

Exercise consisted of 4 × 30 sec maximal running efforts, separated by a 4 min of recovery. Each sprint was performed on a manually driven treadmill (i.e., the subject was the power source for the running belt; Desmo Pro; Woodway^®^; Waukesha, WI) and subjects were required to grip the handle bars to propel the tread with maximal effort. Hemodynamic measures were collected pre and post exercise (~20 min) in 8 subjects.

### Interval sprint training

Exercise training consisted of interval sprint exercise (described above) performed 3 times per week for 6 weeks. The number of sprints increased from 4 to 7 bouts per session, and the duration of each sprint increased from 30 to 45 sec over the 6‐week training program. Hemodynamic measures were collected in the resting condition pre and post training in eight subjects. All training sessions were supervised by a personal trainer ensuring that each session was completed properly.

### Anthropometrics

Body composition (lean mass and fat mass) was determined by whole‐body densitometry using air‐displacement plethysmography (BodPod^®^; Life Measurements, Concord, CA). Testing was performed according to the manufacturer's instructions. Thoracic gas volume was estimated for all subjects, using the predictive equation integral to the BodPod^®^ software. Estimated body density was integrated into the Siri equation (Siri 1961).

### Vascular data collection

Right BA blood pressure was measured by auscultation. Thereafter, beat by beat blood pressure was monitored from the right middle finger by photoplethysmographic methods, and BA pressure was determined via waveform reconstruction (Finometer Model 1, Finapress Medical Systems, Arnheim, Amsterdam, Netherlands). Simultaneously, right BA diameter and blood flow velocity were assessed using ultrasound in a duplex imaging mode (4 MHz Doppler ultrasound; 10 MHz ultrasound imaging; GE Vivid 7; Mississauga, ON, Canada). Stroke volume blood flow velocity was obtained from the ascending aorta (via the suprasternal notch) using pulse wave Doppler (2 MHz pulsed Doppler; GE Vingmed CFM750; Mississauga, ON, Canada).

### Data analyses

Six 2‐D manual caliper measures were taken from a single B‐mode image (10–12 MHz transducer, 27 frames per second, GE Vivid 7) and analyzed offline (EchoPAC Dimension software; GE Healthcare, Baie d'Urfe, Canada – 0.01 mm minimum detectable change), by an experimenter blinded to experimental condition to determine BA lumen diameter during systole and diastole, as well as wall thickness. Analog signals of the instantaneous blood pressure and mean spectral blood flow velocities were collected at 1000 Hz.

### Pulse wave velocity

Pulse wave velocity was calculated as the distance between the suprasternal notch and the middle finger tip divided by the time delay between the foot of the aortic blood velocity waveform and the finger blood pressure waveform. The foot of the pressure wave was identified as the precise point of initiation of the pressure waveform upstroke.

### Brachial artery stiffness indices

Brachial artery vascular properties were analyzed using the indices of arterial stiffness, as described by O'Rourke and Staessen ((O'Rourke et al. 2002); Table 1).

**Table 1:** Vascular mechanic measurements

Variable	Definition
Indices of Brachial Artery Stiffness	
Arterial strain(sD − dD)/dD); expressed as ∆% in D	Amount of deformation relative to the unstressed state
Arterial stiffnessln(Ps/Pd)/(ΔD/Dd); nondimensional	Ratio of logarithm (systolic/diastolic pressures) to (relative change in diameter)
Peterson's elastic modulus(ΔP × Dd)/ΔD; expressed as mmHg	Pressure step required for (theoretical) 100% stretch from resting diameter at fixed vessel length
Arterial distensibilityΔD/(ΔP × Dd); expressed as mmHg^−1^	Relative diameter change for a pressure increment; inverse of elastic modulus
Arterial complianceΔD/ΔP; expressed as cm/mmHg	Absolute diameter change for a given pressure step at fixed vessel length
CLK Model for Forearm Vascular Bed	
Compliance, *C*expressed as mL/mmHg	Elastic deformation of the vascular bed
Inertance, *L*expressed as mmHg/mL/min^2^	Inertia of the blood and vascular bed
Viscoelasticity, *K*expressed as mmHg/mL/min	Opposition to stretch of the vascular bed

Indices of brachial artery stiffness are adapted from O'Rourke and Staessen (O'Rourke et al. 2002).

P, pressure; D, Diameter; s, systolic; d, diastolic.

### Forearm vascular bed mechanics

Mean values of BA blood pressure and flow were averaged over 10 sec to obtain time‐averaged values. Subsequently, continuous waveforms for concurrent time‐aligned BA blood pressure and blood flow were extracted (Lab Chart 7, Powerlab, ADInstruments, Sydney, NSW, Australia) and analyzed using a three‐element lumped Windkessel model (Table 1) as described earlier from our laboratory (Zamir et al. 2009). Briefly, the dynamics of pulsatile flow through a vascular network are influenced by three mechanical properties; C (compliance), K (viscoelasticity), and L (internance). The C, L, and K values are calculated from the oscillatory pressure and flow waveforms at the entry point of the vascular bed (Zamir et al. 2009).

### Statistical analyses

Statistical analyses were performed using Sigma Stat for Windows (version 8.0). Paired two‐tail t‐tests were used to compare hemodynamic as well as, vascular mechanic variables pre and postsprint exercise and training. All data are present as mean ± standard deviation. The significance level was set at *P* ≤ 0.05.

## Results

### Physical characteristics

Age, height, mass, lean mass, and fat mass of all participants are presented in Table 2.

**Table 2:** Physical characteristics

	Age (y)	Height (cm)	Mass (kg)	Lean mass (kg)	Fat mass (kg)
Acute exercise	27 ± 3	181 ± 6	82.4 ± 8.7	68.5 ± 10.1	13.9 ± 4.9
Pretraining	23 ± 6	178 ± 2	82.5 ± 9.2	68.9 ± 5.3	13.7 ± 8.0
Post‐training	NA	NA	82.1 ± 8.5	67.9 ± 5.3	14.3 ± 5.8

### Impact of acute sprint exercise bout

#### Hemodynamics

Twenty minutes following acute exercise, systolic pressure (pre = 116 ± 12 vs. post = 107 ± 13 mmHg; *P* = 0.10) diastolic pressure (pre = 64 ± 7 vs. post = 63 ± 10 mmHg; *P* = 0.83), and mean arterial pressure (pre = 81 ± 8 vs. post = 78 ± 10 mmHg; *P* = 0.36) were the same as pre‐exercise. There was a reduction in pulse pressure following exercise (pre = 52 ± 9 vs. post = 44 ± 9 mmHg; *P* < 0.01). Brachial artery blood flow was increased (pre = 56 ± 33 vs. post = 141 ± 107 mL/min; *P* = 0.02) and forearm vascular R (pre = 1.8 ± 0.8 vs. post = 0.8 ± 0.5 mmHg/mL/min; *P* < 0.01) was decreased 20 min following exercise.

#### Local brachial artery stiffness indices

**Figure 1:**
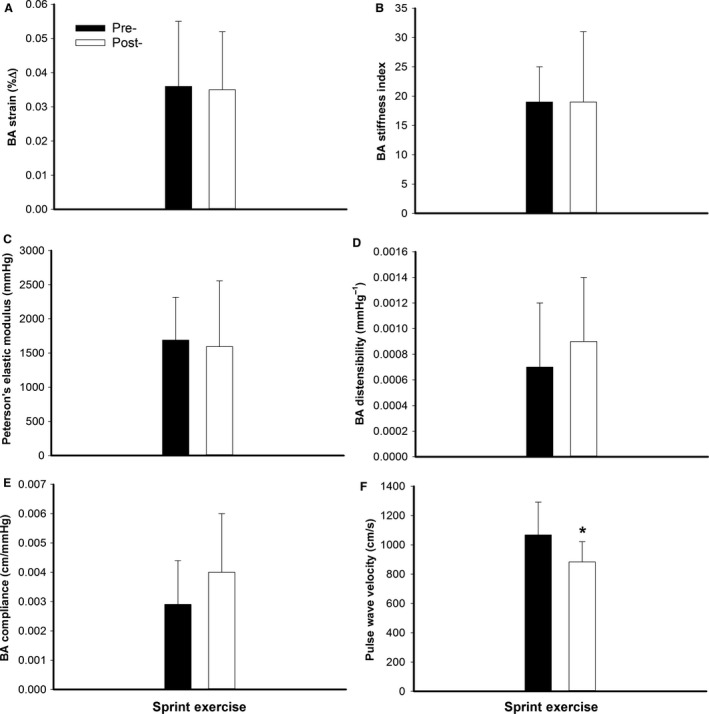
Brachial artery (BA) strain (∆%), stiffness index, Peterson's elastic modulus (mmHg), distensibility (mmHg^−1^), compliance (cm/mmHg), and pulse wave velocity (cm/sec) pre‐ (■; dark bars) and post‐ (□; white bars) interval sprint exercise. *Significantly different than pre‐exercise (*P* < 0.01).

Brachial artery systolic (pre = 4.28 ± 0.39 vs. post = 4.76 ± 051 mm; *P* < 0.01) and diastolic (pre = 4.13 ± 0.42 vs. post = 4.60 ± 0.51 mm; *P* < 0.01) diameters were greater following the acute exercise bout. However, the pulsatile change in BA diameter was not altered by exercise (pre = 0.14 ± 0.06 vs. post = 0.16 ± 0.07 mm; *P* = 0.52). Twenty minutes following acute exercise there were no changes in strain, stiffness index, Peterson's elastic modulus, distensibility, or compliance (*P* ≥ 0.10; Fig. 1A–E).

#### Pulse wave velocity

Compared to the baseline period PWV decreased following the acute exercise bout (*P* < 0.01; Fig. 1F).

#### Forearm vascular mechanics

**Figure 2:**
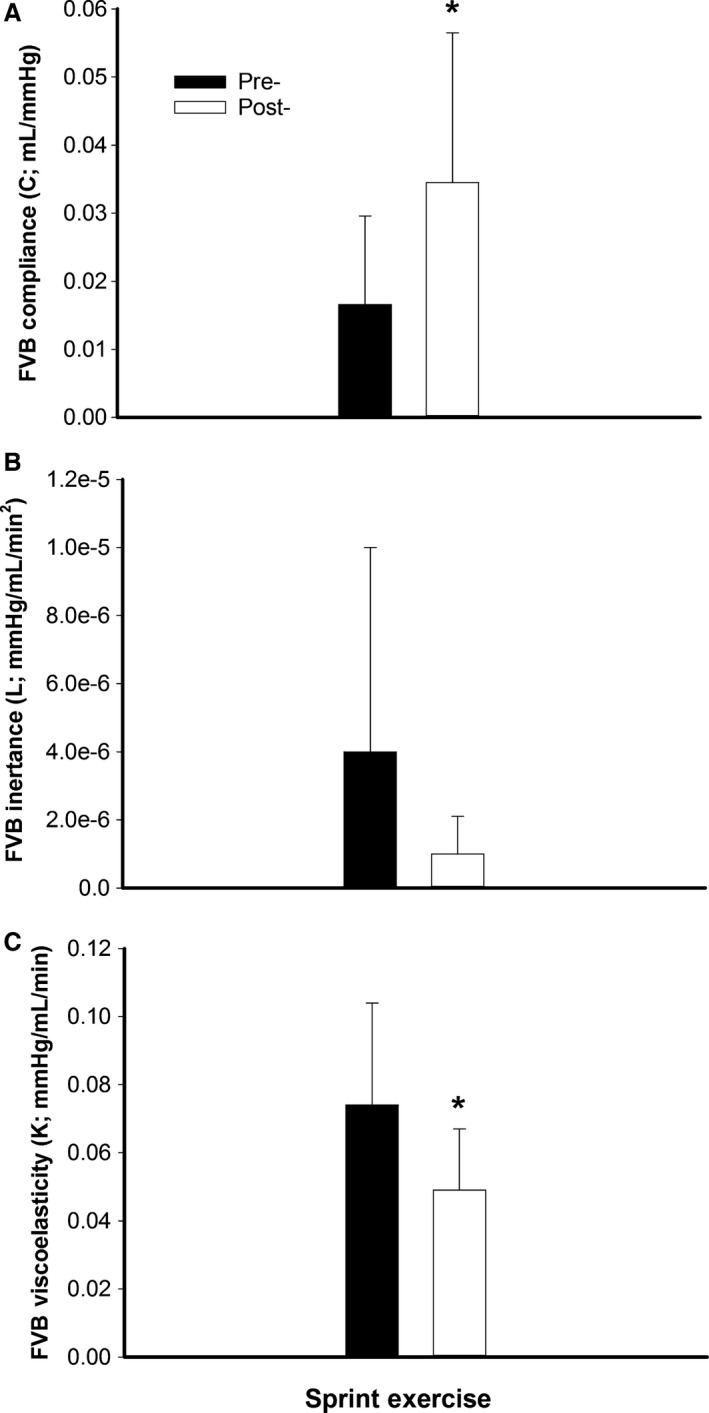
Forearm vascular bed (FVB) compliance (C; compliance; mL/mmHg), inertance (L; mmHg/mL/min^2^), and viscoelasticity (K; mmHg/mL/min) pre‐ (■; dark bars) and post‐ (□; white bars) interval sprint exercise. *Significantly different than pre‐exercise (*P* ≤ 0.03).

Compared to baseline, C increased (*P* = 0.03; Fig. 2A); whereas, L did not change (*P* = 0.15; Fig. 2B) and K declined (*P* = 0.02; Fig. 2C) following the acute exercise bout.

### Impact of six weeks of training

Following chronic training there were no changes in supine baseline systolic (pre = 123 ± 12 vs. post = 122 ± 10 mmHg; *P* = 0.76), diastolic pressure (pre = 66 ± 10 vs. post = 67 ± 10 mmHg; *P* = 0.65) mean (pre = 82.1.5 ± 10.2 vs. post = 80.5 ± 11.9 mmHg; *P* = 0.67) arterial pressure or in pulse pressure (pre = 57 ± 5 vs. post = 54 ± 5 mmHg; *P* = 0.08). Baseline BA blood flow (pre = 42.1 ± 20.7 vs. post = 43.8 ± 14.02 mL/min; *P* = 0.82) and forearm vascular R (pre = 2.4 ± 1.2 vs. post = 2.0 ± 0.7 mmHg/mL/min) did not change with training.

#### Brachial artery stiffness

**Figure 3:**
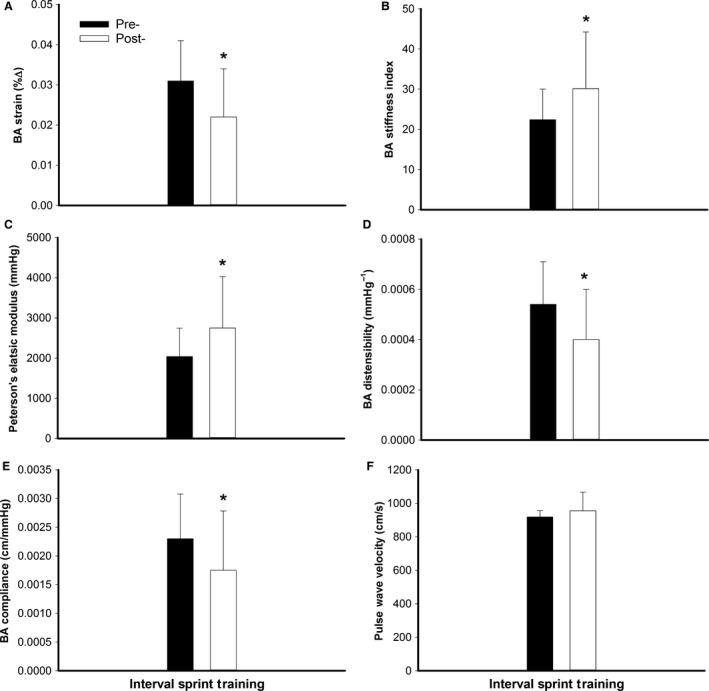
Brachial artery (BA) strain (∆%), stiffness index, Peterson's elastic modulus (mmHg), distensibility (mmHg^−1^), compliance (cm/mmHg), and pulse waved velocity (cm/sec), pre‐ (■; dark bars) and post‐ (□; white bars) interval sprint training.*Significantly different than pre‐training (*P* ≤ 0.05).

Following chronic training there were no changes in BA systolic (pre = 4.37 ± 0.51 vs. post = 4.43 ± 044 mm; *P* = 0.37) and diastolic (pre = 4.24 ± 0.49 vs. post = 4.34 ± 0.42 mm; *P* = 0.18) diameters. However, the pulsatile change in BA diameter declined (pre = 0.13 ± 0.05 vs. post = 0.10 ± 0.05 mm; *P* = 0.03). Compared to pretraining, BA strain, compliance and distensibility declined (*P* ≤ 0.05; Fig. 3A–E), whereas Peterson's elastic modulus and the stiffness index increased (*P* = 0.04; Fig. 3A–E) following training. There were no changes in BA wall thickness following training (pre = 0.34 ± 0.07 vs. post = 0.35 ± 0.12 cm; *P* = 0.53).

#### Pulse wave velocity

Pulse wave velocity did not change with training (*P* = 0.31; Fig. 3F).

#### Forearm vascular bed mechanics

**Figure 4:**
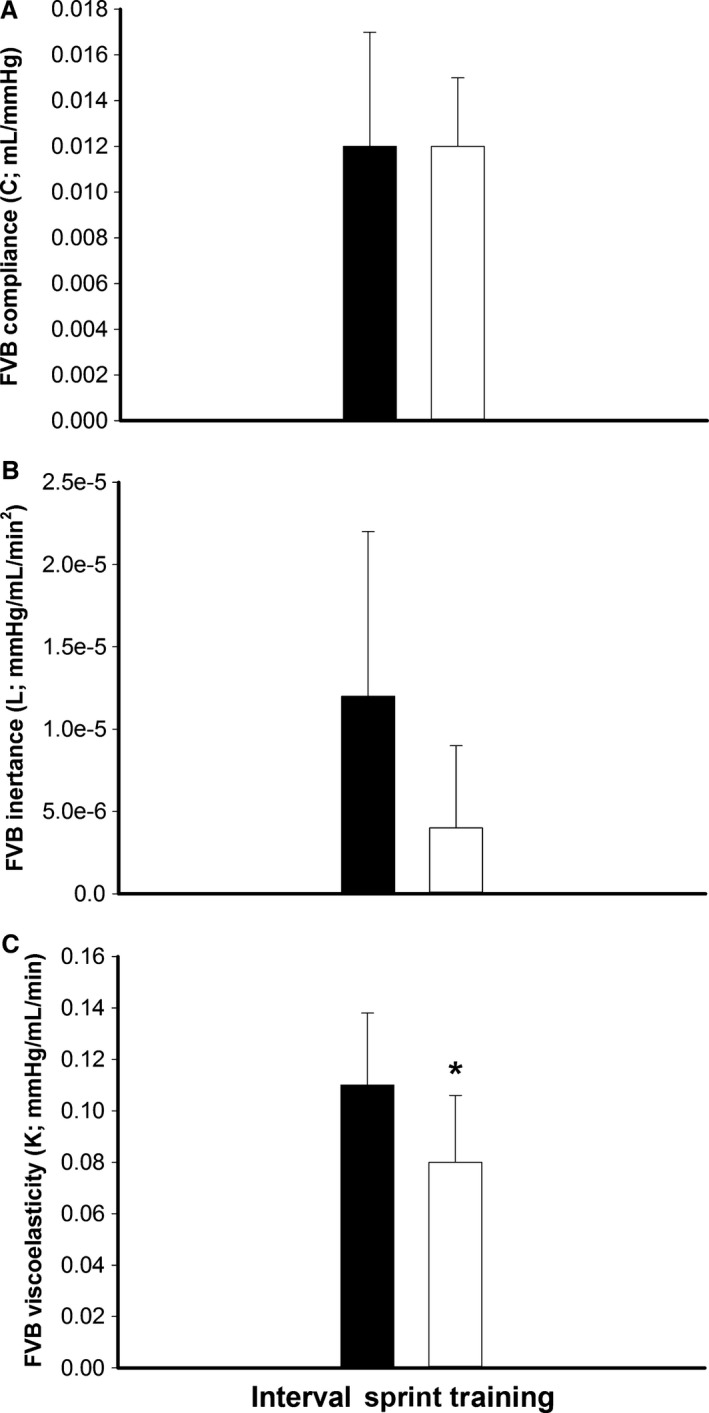
Forearm vascular bed (FVB) compliance (C; compliance; mL/mmHg), inertance (L; mmHg/mL/min^2^) and viscoelasticity (K; mmHg/mL/min) pre‐ (■; dark bars) and post‐ (□; white bars) interval sprint training. *Significantly different than pretraining (*P* = 0.02).

The training did not affect baseline C and L (*P* ≥ 0.10; Fig. 4A and B), but reduced values of forearm vascular K (*P* = 0.02; Fig. 4C).

## Discussion

The major findings of this study were that both acute and chronic interval sprint exercise conducted on a manually propelled treadmill induced changes in vascular mechanical function in the upper limb. Following acute sprint exercise, BA compliance was unchanged; however, PWV declined and there was a shift in forearm vascular bed mechanics, which includes the microvasculature, toward a more compliant system. However, these effects were not carried over with chronic exercise training on a manually propelled treadmill. Specifically, following 6 weeks of training, data suggest that although PWV remained the same, there was a shift toward a stiffer BA. In addition, while forearm vascular resistance (R) and compliance (C) remained unchanged, the viscous opposition to stretch (K) was reduced. Increased BA stiffness and reduced viscous opposition to stretch in the forearm vascular bed suggest that vascular adaptations to interval sprint exercises training on a manually propelled treadmill develop differently along the various segments of the upper limb vascular tree (Table 3).

**Table 3:** Exercise‐induced upper limb vascular mechanical adaptations

Measure/Location	Acute sprint exercise	Interval sprint training
PWV/entire upper limb	↓ stiffness	~
BA mechanics/conduit artery	~	↑ all indices of stiffness
R/Forearm vascular bed	↓ R = ↑ vascular volume	~
C/Forearm vascular bed	↑ C = ↑ elastic ability	~
L/Forearm vascular bed	~	~
K/Forearm vascular bed	↓ K = ↓ opposition to stretch	↓ K = ↓ opposition to stretch

PWV, pulse wave velocity; BA, brachial artery; R, resistance; C, compliance; K, viscoelasticity; L, inertance; ↑, increased; ↓, decreased; ~ and =, unchanged.

### Brachial artery stiffness

Although acute sprint exercise decreased PWV, indicative of decreased upper limb vascular stiffness, indices of BA stiffness remained unchanged. Thus, in contrast to our hypothesis, acute sprint exercise on a manually propelled treadmill did not alter indices of BA stiffness favoring a more compliant conduit artery. Furthermore, interval sprint exercise training on a manually propelled treadmill increased indices of BA stiffness. The divergent response between acute and chronic exercise on BA vascular mechanics indicates that acute exercise‐induced changes such as neural input, shear‐stress or blood‐borne factors released with exercise do not influence BA stiffness acutely. Furthermore, that BA stiffness appears to be unmodified by acute stimuli suggests the changes following training were the cumulative effect of repeated exercise bouts and likely involved remodeling at the level of the BA tissue. The reduction in BA strain, distensibility and compliance following this mode of training is indicative of a reduced arterial deformation (represented by a significant decrease in ∆D) for a given change in blood pressure (O'Rourke et al. 2002). This observation was surprising and perhaps the result of the mode of forearm exercise (isometric, static handgrip), the acute rise in BA blood pressure that occurs immediately following sprint exercise (~2 min) (Rakobowchuk et al. 2009), or perhaps the result of extravascular changes influencing BA vascular mechanics (i.e., altered interstitial fluid content). Increased vascular stiffening may increase left ventricular end‐systolic stress (Arnold et al. 1991); however, this is unlikely an issue in young healthy participants. Furthermore, it is improbable this training has such an effect systemically as the adaptation appears to be localized to the BA. Indeed, carotid artery distensibility does not change and popliteal artery distensibility increases following interval sprint exercise training on a cycle ergometer (Rakobowchuk et al. 2008). Moreover, there was evidence of decreased vascular stiffening in the forearm vascular bed, and consistent with previous results (Cocks et al. 2013; Harris et al. 2014) BA PWV remained unchanged following training. BA C tends to increase up to the sixth decade of life (van der Heijden‐Spek et al. 2000), is increased in hypertension (Izzo et al. 2001) and radial C is increased in obesity (Mangoni et al. 1995). Assuming increased BA stiffness does not induce cardiac stress, the training‐induced reduction in BA C in this study may be protective against age‐, hypertension‐, and obesity‐related changes in upper limb conduit, muscular artery remodeling. Combining our results with those in the literature, interval sprint exercise and training, where the hands grip handle bars and the lower limbs are the prime movers, both appear to improve conduit artery endothelial function in the upper and lower limb (Rakobowchuk et al. 2008; Harris et al. 2014), but this mode of training only increases conduit artery distensibility in the lower limb (Rakobowchuk et al. 2008) and appears to reduce C in the upper limb.

### Forearm vascular mechanics

Twenty minutes after one bout of sprint exercise BA blood flow and forearm vascular C were still greater than at rest and R, K, were reduced. Increased C and decreased K indicate that the elastic ability of the vascular bed increased, and that increase was enabled by a reduced viscous opposition to elastic stretch. These results also suggest that the arterial system was more compliant and the ease at which the system stretched was increased (i.e., increased elastic efficiency). As reductions in R, in this case, indicate an increase in arteriole diameter (not increased vascular volume owing to vascular restructuring), these findings add to our existing understanding of post‐exercise hyperemia. That is, according to the pressure–volume relationship, as fluid volume and arterial diameter increase, the elastic ability of a vessel tends to decrease (Spencer and Denison 1963; Klabunde 2011). However, in this study, arterial diameter and vascular C increased concomitantly following exercise. Potentially, simultaneous increases in vessel diameter and C are mediated through reductions in K, the proportion of viscous opposition to elastic stretch. Interestingly, it has been demonstrated that during sympatho‐excitation, changes in vascular C can operate independently from R (Zamir et al. 2007) and may be adjusted for by changes in K (Zamir et al. 2009). Acute shifts in vascular compliance or the viscous opposition to elastic stretch have not been characterized at the cellular level in these arteries, but may be the result of increased fluid shear‐stress following exercise, and involve changes in ion flux, membrane potential, phosphorylation status and subsequent cross bridge cycling in vascular smooth muscle cells. Alternatively, at the tissue level, vascular smooth muscle cells may rearrange as the vessel dilates; thereby altering the mechanical properties of the vessel (Martinez‐Lemus et al. 2009).

Baseline R and C were unchanged following 6 weeks of exercise training, indicating the forearm arteriole diameter/vascular cross‐sectional area (associated with R) (Zamir et al. 2007), arterial elastic ability (associated with C) (Zamir et al. 2007) and the basal metabolic/neurogenic inputs to R and C likely do not change following interval sprint exercise training on a manually propelled treadmill in healthy young men. Given that R and C were highly responsive to a single bout of exercise, but remained unchanged following training suggests daily exercise or physical activity may be required to sustain these vascular responses. Previously, it was established that normative values for R and C may not exist and that other variables, such as dynamic vascular responsiveness (i.e., the ability to maintain vascular homeostasis to changing flow and pressures under oscillatory conditions), may be the regulated variable in the forearm vascular bed (Zamir et al. 2009). Zamir and colleagues (Zamir et al. 2009) speculated that viscoelasticity (K) may serve as a parameter of control, in that values for K are matched to values of R, C, and L to protect dynamic responsiveness in the vascular network.

In this study, following 6 weeks of exercise training the viscous opposition to elastic stretch (K) decreased, though vascular C itself did not change. This was among the few adaptations that was similar following acute and chronic exercise and suggests K is both highly responsive and perhaps more revealing of changes in vasomotor control of pulsatile flow than R or C alone (Zamir et al. 2009). At the tissue level, it has been speculated that a chronic reduction in K indicates a shift in the collagen:elastin ratio, changes in smooth muscle or elastin architecture, or changes in the polysaccharide and connective tissue matrix surrounding vascular smooth muscle cells (Zamir et al. 2009). Mechanical signals, perhaps those experienced during interval sprint exercise, can mediate changes in cytoskeletal protein expression that alter the tensile state of vascular smooth muscle cells and impact force transmission; thereby, altering vascular wall mechanics (Martinez‐Lemus et al. 2009). Experimental evidence suggests K is elevated in smokers versus nonsmokers (Nielson et al. 2014) and in old versus young participants (Zamir et al. 2009). Furthermore, a lifestyle intervention involving nicotine replacement therapy and moderate intensity exercise intervention did not reduce K in smokers (Nielson et al. 2014). In this respect, interval sprint exercise training on a manually propelled treadmill may help prevent age‐ and pathological‐related changes (i.e., increased K resulting in decreased elastic efficiency) in the forearm vascular bed, but future research is required to address this possibility.

### Significance

In this study, training‐induced vascular adaptations were heterogeneous along the vascular tree of the upper limb and did not favor a systemic increase in upper limb vascular compliance. Following training the BA displayed reduced deformation for a given change in blood pressure, indicating a reduction in segmental conduit vessel compliance. Conversely, reductions in K indicate the ability to impede stretch across the forearm vascular bed was decreased. In spite of adaptations occurring in the BA and forearm vascular bed, total upper limb vascular stiffness remained unchanged. Thus, the nature of the vascular adaptations induced by interval sprint exercise training on a manually propelled treadmill is heterogeneous along the vascular tree in the upper limb. Furthermore, when compared with work from Rakobowchuk and colleagues (Rakobowchuk et al. 2008) (performed on a cycle ergometer), these data suggest structural adaptations in conduit arteries are different in the femoral artery versus BA following interval sprint exercises training. This may be owing to the fact that this type of exercise training consists of isotonic, dynamic, lower limb versus isometric, static, upper limb skeletal muscle contractions and as a result arteries are likely exposed to different fluid shear patterns. These observations confirm a breadth of research, predominantly in animal models (Armstrong and Laughlin 1984; Musch et al. 1987; Laughlin et al. 1988, 1996, 2006, 2008, 2012; Sexton and Laughlin 1994; Lash and Bohlen 1997; Laughlin and Roseguini 2008), indicating that the degree of, and spatial distribution of training‐induced vascular adaptations are different following different modes and intensities of training (Armstrong and Laughlin 1984; Musch et al. 1987; Laughlin et al. 1988, 1996; Sexton and Laughlin 1994), and are distributed nonuniformly along the arterial tree (Lash and Bohlen 1997; Laughlin et al. 2006, 2008, 2012; Laughlin and Roseguini 2008).

### Limitations

The modest sample size in this study may have reduced statistical power for detecting acute and chronic exercise‐induced changes in select variables. For example, analyses of Cohen's D effect sizes ((Post_mean_ − Pre_mean_)/grouped SD)(Cohen 1988) on variables with *P* values between 0.05 and 0.10 reveal that acute exercise had a medium effect on systolic blood pressure and BA C, and chronic training had a medium and large effect on pulse pressure and forearm vascular L, respectively. Acute reductions in systolic blood pressure and chronic decrease in pulse pressure highlight the potential therapeutic efficacy of this exercise modality in the treatment of hypertension and cardiovascular disease risk, but future research in clinical populations is required to confirm this possibility. Importantly, these observations do not alter the overall conclusions of the study, particularly that following 6 weeks of training vascular adaptations are heterogeneous along the vascular tree, indicated by increased stiffness in the BA and evidence of decreased stiffness in the forearm vasculature.

In this study, the intensity of handgrip was not controlled and was unlikely to be the same across bouts within or between participants. It is unlikely that either stimulus (sprinting or static handgrip) was solely responsible for the changes in BA and forearm vascular mechanics. Both lower body and handgrip exercise increase forearm blood flow in an intensity‐dependent manner, but operate through different mechanisms (Green et al. 2005). Furthermore, in this study forearm blood flow was elevated ~20 min post‐exercise whereas, following handgrip exercise alone, forearm blood flow values typically return to baseline levels within a few minutes after exercise cessation (Byström and Kilbom 1990; Kagaya and Homma 1997; Hicks et al. 1999; VanBeekvelt et al. 2001; Osada et al. 2003). Thus, the alterations in BA and forearm vascular mechanics were likely caused by the combination of sprinting with concurrent static handgrip.

Also, the models used to assess vascular remodeling utilize different parameters, rendering it difficult to compare results directly. Nevertheless, there is a degree of overlap in the mechanical properties assessed by each model and each model examines vascular remodeling at different locations along the arterial tree. For this reason, combining models may be superior to relying on a single model when examining vascular remodeling in the entire upper limb.

### Perspectives

Interval sprint exercise performed on a manually driven treadmill causes an acute shift in forearm vascular mechanical function toward a more compliant vascular bed without concurrent changes in BA compliance. Chronic adaptations to interval sprint exercise training on a manually propelled treadmill in the forearm vasculature are indicative of increased elastic efficiency, without changes in absolute elasticity. In contrast to the forearm vascular bed, indices of BA stiffness favored a stiffer artery post‐training. Therefore, acute and chronic vascular adaptations were not directionally consistent with one another and vascular adaptations induced by interval sprint exercise training on a manually propelled treadmill were not uniform along the arterial tree of the upper limb.
